# Bridging the gap: Evidence-based practice guidelines for sports nutritionists

**DOI:** 10.3389/fnut.2023.1118547

**Published:** 2023-03-29

**Authors:** Alex J. Ritson, Mark A. Hearris, Laurent G. Bannock

**Affiliations:** ^1^The Institute of Performance Nutrition, Edinburgh, United Kingdom; ^2^Department of Sport and Exercise Sciences, Manchester Metropolitan University, Manchester, United Kingdom

**Keywords:** sports nutrition, science-to-practice, decision-making, nutrition intervention, personalised nutrition, athlete

## Abstract

Evidence-based practice is a systematic approach to decision-making developed in the 1990s to help healthcare professionals identify and use the best available evidence to guide clinical practice and patient outcomes amid a plethora of information in often challenging, time-constrained circumstances. Today’s sports nutrition practitioners face similar challenges, as they must assess and judge the quality of evidence and its appropriateness to their athlete, in the often chaotic, time-pressed environment of professional sport. To this end, we present an adapted version of the evidence-based framework to support practitioners in navigating their way through the deluge of available information and guide their recommendations to athletes whilst also reflecting on their practice experience and skills as evidence-based practitioners, thus, helping to bridge the gap between science and practice in sport and exercise nutrition.

## Introduction

The advancement of sport and exercise nutrition (SEN) as a recognised sub-discipline of sport and exercise science (SES) began in the late 1960s, following the pioneering work of Scandinavian scientists who investigated the role of muscle glycogen and carbohydrate availability on exercise capacity and performance (1–4). More than 50 years on, the application of sports nutrition research is now recognised as paramount to the wellbeing and performance of athletes (5). The growing recognition and interest among governing bodies, the academic community, and the exercising public have led to a substantial rise in SEN publications (a 35-fold increase in published papers over the past three decades) (6, 7), with a concomitant rise in practitioners working in elite sport (8).

Practitioners must be well-versed in the four rapidly evolving disciplines that comprise SEN: exercise biochemistry and integrated metabolism, exercise physiology, nutrition, and psychology (9). Moreover, practitioners must translate their knowledge of these disciplines into practical nutrition strategies that assist their athletes in modulating their capacity to meet the key performance determinants aligned with their sport (5). In contrast to the reality in which practitioners cross and integrate a variety of disciplinary boundaries to deliver their nutrition and lifestyle strategies, the science from which this information is derived is typically studied as separate disciplines. Furthermore, such studies are often undertaken on mostly male populations, with ill-defined significance to the professional athlete, using non-ecologically relevant training models, and outside of the “real-world,” complex, and often chaotic environment of professional sport (10–12). Recent publications have drawn attention to a significant body of sport and exercise research (including nutrition) that lacks translation to practice due to study design quality and relevance (8, 13–15). Despite the steadily growing body of scientific knowledge in SEN, there exists an even greater amount of noise in the form of “scienciness” (16) that is widely available through internet-based information and social media, further widening the gap between quality and flawed knowledge for the practitioner to navigate (17).

While SEN research is being published at a *faster* rate, with a *higher* proportion of investigations each year, journals are demanding *stronger* scientific rigour in research reporting (e.g., the PRESENT 2020 checklist) to improve the quality of research in the long term (18); collectively in keeping with the Olympic Games motto *Citius, Altius, Fortius*—which is Latin for “Faster, Higher, Stronger.” Furthermore, more ecologically valid studies on athletes are being conducted due to increased scientific acceptance by governing bodies, in addition to technological progress and breakthroughs (7). Thus, the science of SEN, like the athletes it seeks to inform, is proactively improving. The modern-day practitioner is, therefore, entering an exciting period of SEN science, which should look to better serve those on the front line in the future.

With that said, today’s practitioners are and will continue to be faced with the challenge of keeping up with the now rapidly evolving and expanding body of knowledge in SEN, while evaluating its validity and relevance to the unique context of the athlete. In accordance with this, the practitioner should regularly reflect on their practice experiences and professional competencies to provide the best possible care to their athletes and support researchers in identifying practically relevant areas of future study. Therefore, we propose that the practitioner requires an evidence-based framework to enable and support an efficient and effective information-sifting process, to select, translate and apply the most appropriate evidence to their athletes, whilst pro-actively reflecting on their experiences and refining their skills as practitioners, thus *bridging the gap between science and practice*; and in doing so, improving the standard of applied SEN.

## Evidence-based practice

In the 1990s, a group of physicians at McMaster University in Hamilton, Canada, pioneered “evidence-based” medicine, foreshadowing evidence-based practice (EBP). It has been described as one of modern medicine’s paradigm shifts (19), providing a framework for practitioners to blend the best available evidence with professional reasoning whilst dealing with an excessive amount of information within an often severely limited time as a healthcare professional. Most importantly, the EBP framework triangulates evidence and judgement with patient preferences, values, and circumstances, with its appropriateness determined within the context in which it is to be applied rather than a “one-size-fits-all” solution (20). In medicine, EBP has been defined as “integrating individual clinical expertise with the best available external clinical evidence from systematic research” (21).

In keeping with recent definitions of EBP in other domains of SES (22), we propose the following EBP definition for SEN: “A thorough, integrated approach to the nutritional support of athletes based on the best available evidence and expert professional judgment, taking into account the athlete’s key performance determinants, values, goals, personal preferences and circumstances, as well as the practitioner’s work context.” The terms “thorough” and “integrated” refer to a methodical, systematic approach to identifying the best, most relevant evidence from the integration of SEN disciplines. This process is constantly evolving, and strategies will change as new research and modes of knowledge production are established. “The best evidence” refers to retrieving the most relevant, peer-reviewed research, grey literature (evidence derived outside commercial or academic publishing), and professional experience to address the problem at hand. The term “professional judgment” acknowledges the sporting culture of the athlete, physiological, metabolic, nutritional, environmental, and commercial demands as a professional, previous experience with nutritional strategies, and their circumstances and values. “Work context” refers to the working environment in which the practitioner finds themselves, which will determine how they interact with the athlete and deliver their nutritional strategies.

Evidence-based practice is commonly described as a five-step process, which we have adapted to meet the specific demands of the SEN practitioner. 1: *Ask* – translate athlete information into answerable outcome-based questions. 2: *Acquire* – find the best available evidence to answer the question. 3: *Appraise* – evaluate the evidence critically for validity, relevance, and applicability. 4: *Apply* – integrate evidence, context, and practice into professional decision-making and recommendations. 5: *Audit* – reflect and evaluate processes 1 through 4 and seek ways to continually improve.

**FIGURE 1:**
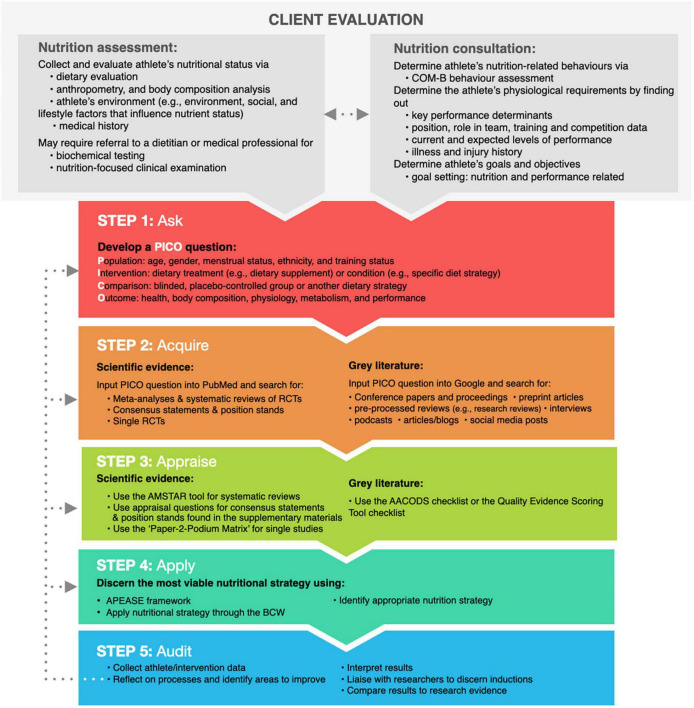
An adapted overview of the five-step EBP framework for sports nutrition practitioners. The dotted lines illustrate self-auditing Steps 1–4 to identify opportunities for development using the EBP framework. BCW, behaviour change wheel; RCT, randomised controlled trial.

The EBP framework for healthcare professionals, according to Straus et al., can be used in three areas of medicine: diagnosis, prognosis, and intervention (23). While identifying a nutritional diagnosis (e.g., iron deficiency anaemia) is the second of the four key steps in the Nutrition Care Process for a sports nutritionist (24), it may be outside the SEN practitioner’s scope of practice, requiring either specialist clinical credentials (e.g., RD and MD), or a professional referral to an appropriate specialist. Therefore, to extend our reach to all qualified sports nutritionists (the practitioner), the EBP framework presented herein (and shown in Figure 1) will centre around the implementation of nutritional strategies for athletes (e.g., a nutritional intervention), being the third step in the Nutrition Care Process.

### Step 1: Ask

Before implementing a nutritional intervention with an athlete, a nutrition assessment and consultation are required. A nutrition assessment may include an evaluation of the athlete’s diet, anthropometry and body composition, biochemical testing, a nutrition-focused clinical examination, and an audit of the athlete’s medical history (24). An in-depth overview of a nutrition assessment is beyond the scope of this review; however, interested readers should review a thorough overview of this process by Larson-Meyer et al. (25). A nutrition consultation allows the practitioner to better understand the athlete’s behaviours, preferences, training demands, illness and injury history, current challenges and projected nutrition, and performance-related goals. Based on the information gathered, the practitioner may discover they do not have all the answers to support their athlete. A practitioner’s uncertainty should be the starting point for using the EBP framework.

The first step in gathering the best evidence for use in practice is to ask a question. A well-crafted question is required to ensure the practitioner does not waste time searching for the best available evidence. With the amount of information available expanding exponentially, finding the most appropriate evidence to address the athlete’s issue requires the practitioner to develop a clear understanding of the athlete’s needs and requirements (26), using foreground questions in conjunction with the PICO method described below.

A practitioner’s inquiry can begin with background or foreground questions. Background questions are designed to elicit general information about the context of a situation, condition, state, or another aspect of SEN, and must be answered before posing a foreground question. Depending on a practitioner’s academic training, competent practitioners should be able to answer most background questions. Therefore, this review will concentrate on foreground questions, which focus on specific knowledge that gathers the best evidence to solve an athlete’s issue. The EBP framework can use the PICO model to generate foreground questions, which stands for “population,” “intervention,” “counter-intervention,” and “outcome.”

#### Population

The practitioner must be explicit about the characteristics of the population to which their question refers. The question may want to consider some of the following information: age (e.g., adolescent, young, middle-aged, and older), gender, menstrual status (e.g., regularly menstruating and contraceptive use), ethnicity, and training status, as each have the potential to influence the relevance of the evidence required to inform their nutritional intervention (12, 27–30).

#### Intervention or issue of interest

The practitioner must be specific about the main intervention, independent factor, or variable in their question. For this review, the intervention is focused on a dietary treatment (e.g., provision of a dietary supplement) or condition (e.g., a hypoenergetic diet); however, other examples include, but are not limited to, an exposure (e.g., environment and exercise test), or issue in which the practitioner is interested (e.g., leg immobilisation)—the more specific the description of the intervention, the narrower the search.

#### Counter-intervention (optional)

When formulating a question, the practitioner may include an alternative to compare to the intervention; however, this is not always necessary. For example, if the intervention is a dietary supplement, the comparison could be a no-treatment group masked by using a placebo or a blinded control group; however, this would not be implied in the question. Using the practitioner’s background knowledge to develop their question, the comparison could be a common dietary practice used in this context or the “standard” approach. A practitioner, for example, might want to assess the outcomes of a popular dietary treatment (“intervention,” e.g., a low-carbohydrate high-fat diet) versus the common dietary approach (“counter-intervention,” e.g., high-carbohydrate diet) on key performance outcomes for their athlete (see Table 1).

**TABLE 1:** An example of creating a foreground question using the PICO model in SEN.

Background question			
A question that aims to find out general information about some aspect of athlete care.			
**Example background question**			
What are the key performance determinants for a marathon runner?			
**Foreground question**			
A question that aims to elucidate specific evidence to inform decision-making. Foreground questions can use the PICO acronym to help narrow and define the question (as shown below).			
**PICO structure**			
Who is the **population** of interest?	What is the **intervention** of interest?	What is the **counter-intervention** (optional)?	What is the **outcome(s)** of interest?
In _____(P)	How does _____(I)	Compared with _____(C) (optional)	Affect _____(O)
**Example foreground question**			
In endurance athletes (P), how does a high-fat diet (I) compared to a high-carbohydrate diet (C) affect performance (O)?			
**PICO question**			
In endurance athletes, how does a high-fat diet compared to a high-carbohydrate diet affect performance? A systematic breakdown of creating a searchable question in PubMed using the above example is shown below: Concept 1: Endurance athlete **(population)** Search terms: “Athletes”[MeSH] OR “athlete*”[tw] Concept 2: High-fat diet **(intervention)** Search terms: “Diet, high-fat”[MeSH] OR “diet, ketogenic”[MeSH] OR “diet, carbohydrate-restricted” [MeSH] OR “high fat”[tw] OR “keto*”[tw] OR “low carb*”[tw] Concept 3: High-carbohydrate diet **(comparison)** Search terms: “Dietary carbohydrates”[MeSH] OR “high carb*”[tw] Concept 4: Performance **(outcome)** Search terms: “Physical endurance”[MeSH] OR “athletic performance”[tw] OR “physical endurance”[tw] **Question to search in PubMed (add all search terms together):** “Athletes”[MeSH] OR “athlete*”[tw] AND “diet, high-fat”[MeSH] OR “diet, ketogenic”[MeSH] OR “diet, carbohydrate-restricted” OR “high fat”[tw] OR “keto*”[tw] OR “low carb*”[tw] AND “dietary carbohydrates”[MeSH] OR “high carb*”[tw] AND “physical endurance”[MeSH] OR athletic performance [tw] OR physical endurance [tw] **Question to search using Google (grey literature) or Google Scholar (scientific research):** Athlete*AND high-fat diet OR ketogenic diet OR low carb* AND carbohydrate diet OR high carb* AND endurance performance or athletic performance			

MeSH stands for “Medical Subject Headings”, which are key terms developed by the National Library of Medicine to index and label research articles in PubMed. Text Word (tw) refers to all words or numbers in the title, abstract, or text of the article. An asterisk (*) at the root of a word is used to find multiple word endings (e.g., carb, carbohydrate, and carbohydrates). Wildcards (not shown above) are symbols that replace a character within a word, such as a question mark (?), to ensure that different spellings of a word are captured (e.g., “wom?n” would gather results containing both “woman” and “women”). Boolean operators are words used to combine search terms. The main Boolean operators are “OR”, “AND”, and “NOT”. “OR” is used to separate similar keywords such as synonyms, acronyms, or spelling variations, “AND” is used to connect ideas or concepts, “NOT” (not shown above) is used to exclude keywords from the search. MeSH and tw functions are only required for searching for literature within PubMed.

#### Outcome

The outcome to a question refers to what the practitioner is trying to achieve, measure, improve, or affect. A practitioner’s question may include multiple outcomes. Suppose all the outcomes are not included in a single study; in that case, the practitioner may need to formulate several questions, change the question’s outcome each time, and evaluate more evidence. For example, a practitioner may want to know how a diet intervention impacts performance, metabolism, and its associated side effects. Although these outcomes may be included in the same study, the practitioner may need to search across multiple studies to find the appropriate evidence.

Table 1 provides an overview of formulating a PICO question for scientific literature using the PubMed database and grey literature using the Google search engine. After developing a specific question, the next step is acquiring the best-available evidence to answer the question.

### Step 2: Acquire

Once the PICO question has been developed, the practitioner should consider acquiring the best available scientific literature as their foundation and triangulating this information with relevant grey literature and professional experience. The quality of scientific evidence often relates to its susceptibility to bias based on study design and is commonly organised into tiers of evidence (see Table 2). For intervention-focused questions, the preferred forms of evidence are systematic reviews and meta-analyses of randomised controlled trials (RCTs) (Level 1) and evidence syntheses and guidelines (e.g., position stands and consensus statements) (Level 2) due to their rigorous critical appraisal process. Although Level 1 and Level 2 evidence are considered the most credible sources of scientific evidence, in the context of applied SEN, this is not a hard and fast rule. Given the systematic rigour or large-scale collaborations associated with such publications, these articles can take years to publish, which could mean their findings have been surpassed by more recent research. Moreover, the review may not cover all components of the practitioner’s PICO question in sufficient detail, which means they may need to acquire more evidence *via* lower hierarchy tiers, starting with un-pooled, well-designed RCTs (Level 3). While well-designed RCTs provide a level of robustness for internal validity and thus increased certainty that the outcomes are caused by the intervention, due to the high levels of controls implemented, this may reduce the practical relevance of the results. If there is insufficient evidence to fully answer a practitioner’s question using Level 1–3 evidence, the practitioner may need to consult other lower-tier evidence such as less well-controlled trials and observational research (Level 4), case studies/reports (Level 5), and “expert opinion” (Level 6). When considering the effectiveness of an intervention, caution should be used when evaluating lower tiers of evidence due to the lack of controls and the potential for bias. However, they can provide practical insights that are often unattainable with well-controlled studies, such as undertaking more extended intervention periods (e.g., a cohort study) or studying an intervention within its real-world context (e.g., a case study on an Olympic athlete).

**TABLE 2:** Levels of evidence for intervention-focused questions and their implications for practice.

Level	Study type	Definition	Implications for practice
1	Systematic reviews and meta-analyses of RCTs	Evidence comes from well-designed and applicable RCTs (e.g., high translational potential), which demonstrate consistent findings across multiple studies.	*Strong recommendation:* Practitioners can provide strong recommendations unless a clear and compelling rationale for an alternative approach is present.
2	Position stands and consensus statements	Evidence comes from the best available, peer-reviewed evidence determined by an independent, often multidisciplinary, panel of experts for the purpose of advancing the understanding of a specific issue, procedure, or method.	*Strong recommendation:* Practitioners can provide strong recommendations unless a clear and compelling rationale for an alternative approach is present.
3	Randomised controlled trials (RCTs)	Evidence comes from single RCTs or multiple RCTs where findings are inconsistent.	*Recommendation:* Practitioners can provide recommendations but should remain open to new evidence that becomes available.
4	Less well-controlled trials and observational research	Evidence comes from poorly controlled trials or uncontrolled observational studies.	*Optional:* Practitioners should be flexible in their decision-making regarding the application of such interventions and may consider alternatives.
5	Case studies/reports	Evidence comes from single case studies and reports.	*Optional:* Practitioners should be flexible in their decision-making regarding the application of such interventions and may consider alternatives.
6	Expert opinion	Expert opinion comes from professional experience or knowledge that does not meet the above criteria.	*Optional:* Practitioners should consider all options in the decision-making process and remain open to new evidence to clarify the risk versus reward. Athlete preference may have a substantial influencing role in the decision-making process.

RCT, randomised controlled trial.

Grey literature is used to describe all materials and research that are produced outside of commercial and academic publishing channels and encompasses evidence derived from pre-appraised research such as research reviews, reputable sports nutrition organisation websites, podcasts, blogs, conference proceedings, and social media posts (31). When led by researchers and research-active practitioners, grey literature such as research reviews, podcasts, blogs, and Twitter threads provide practitioners with rapid access to information on the latest publications, emerging research areas before publication, or field insights that would have previously remained within small peer groups. Unlike scientific literature, however, grey literature may not be peer-reviewed or appraised by experts on the covered topic, increasing the possibility of error and bias.

Professional experience refers to knowledge gained from working with athletes and within specific professional settings (e.g., sporting organisations). Although experience-based anecdotes from the field generally are the least objective and most susceptible to bias (32), there are quality levels similar to that applied to scientific evidence when it comes to the degree of confidence that can be placed on information derived from practical experience. We believe that insights from professional experience carry the most weight when the practitioner is proactively collecting high-quality data and reflecting on their actions and outcomes (see Step 5). Indeed, measurements of body composition (e.g., surface anthropometry), aspects of metabolism (e.g., substrate utilisation), and performance indicators (e.g., lactate threshold) are becoming increasingly accessible to practitioners in the field. Assuming the practitioner upholds the highest possible standards when gathering and managing data over time and that the data is assessed with an understanding of the fundamental principles and latest scientific evidence of SEN; then it would not be unreasonable to treat their practice-based experience and insights with the same level of value as Level 5 evidence.

The methods used to search for scientific and grey literature are similar and typically consist of three key steps (31). Before beginning any search, the practitioner should refine their search parameters based on the key search terms in their PICO question (Step 1). Both search methodologies utilise truncation and wildcard, phrase searching in quotes, and Boolean operators to help pinpoint the specific evidence relevant to the question (31). Another factor to consider when looking for scientific literature is the use of specialised subject headings (MeSH), which aim to capture variations of keyword search terms used in previous research. Once established, the search process can start by identifying key databases (e.g., PubMed for scientific publications) and search engines (e.g., Google for grey literature) (Step 2). The final phase involves screening search results to identify evidence for final selection and appraisal (Step 3). The practitioner should consider recording their search strategies, specifying the period (e.g., 2010–2023) searched and methodological filters employed (e.g., systematic review), and organising their evidence into a bibliographic management tool to improve their efficiency in locating and updating evidence on a particular topic (23). Furthermore, practitioners can keep abreast of components of their PICO question by following the first and last authors of high-quality papers on the topic *via* platforms such as ResearchGate and, in some cases, Twitter.

### Step 3: Appraise

Once all the available evidence has been collected, it must be appraised by the practitioner before being used to inform the decision-making process. This is more important than ever given the increasing availability of scientific publications, which can vary in terms of scientific rigour, as well as the increased accessibility of non-scientific opinions and anecdotal evidence, fuelled by the growth of social media platforms and alternative media (e.g., podcasts). When evaluating research findings, the hierarchies of evidence framework can be used to rank each piece of evidence based on its probability of bias, providing the practitioner with a measure of certainty that can be placed in their recommendations (see Table 2). As a result, practitioners are advised to prioritise the use of high-level evidence to inform their recommendations but should not overlook the use of lower-tier evidence when higher-level evidence is insufficient.

Although high-level evidence is considered less susceptible to bias, the practitioner should still critically appraise the quality of the data and the appropriateness of this evidence to their PICO question. For example, systematic reviews can be evaluated using the AMSTAR tool (33), which can assist practitioners in identifying high-quality, high-level evidence to support their decision-making. When available, the practitioner is also encouraged to refer to separate sub-group analyses within such reviews to identify the available studies relevant to their specific PICO question. If this is not possible, it is recommended that relevant, individual RCTs be critically evaluated. When appraising evidence-synthesis guidelines (e.g., position statements and consensus statements), practitioners should assess if new evidence has become available since publication to ensure their recommendations are based on the most recent evidence (additional appraisal suggestions for evidence-synthesis guidelines can be found in Supplementary material). In critically appraising individual RCTs and other lower-level single studies, the recently developed “Paper-2-Podium Matrix” framework provides the practitioner with a systematic, time-efficient evaluation tool to assess the appropriateness of SEN research (8). This nine-point operational framework allows the practitioner to evaluate the level of scientific rigour, the appropriateness of the methodology used and the feasibility of application to determine the translational potential of the data.

While the hierarchical approach to evidence appraisal prioritises high-level evidence, practitioners are encouraged not to overlook lower-tier evidence, which may provide important evidence of the appropriateness, likelihood of acceptance, and feasibility that is often overlooked or difficult to implement in highly controlled RCTs. For example, although case studies are seen as lower-tier evidence due to their low levels of control, they provide important insight into the feasibility and practical application of specific interventions and often provide unique insight into highly niche population groups such as elite athletes. Using the work of Stellingwerff (34) as an example, this case study demonstrates the practical application of low carbohydrate periodisation strategies within three male marathon runners and provides the practitioner with invaluable information relating to its feasibility and appropriateness within truly elite athletes (34).

Given that evidence from grey literature often bypasses traditional peer-review processes, the quality of this evidence will vary greatly and is highly susceptible to bias. As a result, a thorough appraisal of grey literature is critical before this evidence is used to inform the decision-making process. The AACODS checklist (35) and Quality Evidence Scoring Tool (36) offer practitioners two critical appraisal methods to evaluate grey literature. Finally, evidence from professional experience should be evaluated by determining the approaches used in practice (methodical, reflective, or random), their academic background, and their scientific understanding, which can also be appraised using the tools for appraising grey literature mentioned above.

### Step 4: Apply

After acquiring and appraising the available evidence relevant to their question, the practitioner’s decision to apply the evidence must be guided by their professional experience and understanding of the athlete (37). According to the original definition by Sackett et al., it should be the “conscientious, explicit, and judicious use” of evidence to inform the individual’s care (21). This notion suggests that the practitioner is not required to use the evidence precisely as it was obtained; instead, it can be applied in the manner that best corresponds to the athlete’s context (38). This is where we believe the art of coaching remains firmly preserved within the framework of EBP.

Field expertise refers to understanding the context of the environment or organisation where the intervention will be applied (39). The practitioner often works as part of a multi-disciplinary team of professionals in an organisation or private practice with athletes and their support personnel. The practitioner’s expertise will assist them in comprehending how, within the athlete’s environment and organisational culture, each stakeholder (e.g., chef, coaches, and medical) can influence the application of the evidence and how they can collaborate to facilitate the nutritional intervention. Thus, essential qualities for the practitioner include being aware of the roles and responsibilities of stakeholders working to support the athlete, developing relationships and trust within this realm, and collaborative and communication skills.

The practitioner faces the challenge of communicating their knowledge into practical health and performance solutions for athletes and key stakeholders with varying levels of sports nutrition knowledge and availability to receive information. This necessitates adjusting the delivery of knowledge based on the individual and scenario. Although intended for sports scientists, the knowledge translation framework developed by Bartlett and Drust serves as an important tool for assisting the practitioner in effectively communicating their recommendations to athletes and relevant stakeholders (40). We believe the skill of knowledge translation and the ability to build trust with athletes and relevant stakeholders enables practitioners to develop from “competent” to “proficient.”

Understanding the athlete requires a consideration of their needs, values, preferences, behaviours, and adjusting the nutritional strategy to accommodate these aspects. When implementing any nutritional strategy, the practitioner must keep in mind that the activity is carried out primarily through a set of behaviours that are formed by, among other factors, the athlete’s unique characteristics outlined above. According to Michie et al. (41), all behaviours stem from a combination of the following three components: Capability (psychological and physical), Motivation (reflective and automatic), and Opportunity (physical and social environment), known as the COM-B model of behaviour change (41). Various behaviour frameworks have been merged to develop the Behaviour Change Wheel (BCW) (42), which is centred on the COM-B paradigm and provides a series of intervention functions and policies to assist with the effective implementation of behaviour change.

Michie et al. (41) recommend that practitioners follow a series of steps to assist in understanding their client’s target behaviour (Step 1), identify the most effective intervention(s) and policy categories through which the behaviour can be applied (Step 2), and determine which behavioural change techniques and mode of delivery best support effective change (Step 3). Once the target behaviour(s) to facilitate the nutritional intervention have been selected (Step 1), the practitioner should determine what needs to change in the athlete and their environment to bring about the desired change in behaviour. This can only be accomplished by recognising each athlete as an individual and working through the COM-B model components that may influence their aptitude to implement the nutritional strategy (see Supplementary material for an example). Once the practitioner has identified intervention functions, policies, and techniques to facilitate the nutritional strategy, they should assess the applicability of their proposed intervention, policies, and techniques against a checklist of six criteria: Affordability, Practicability, Effectiveness, Acceptability, Side Effects/Safety, and Equity (APEASE) (see Table 3).

**TABLE 3:** APEASE checklist for selected intervention functions, policies, and behaviour change techniques.

APEASE checklist	Example questions
Affordability	Is the intervention within the athlete or organisation’s budget?
Practicability	Can the intervention be delivered in the manner intended?
Effectiveness	Is the intervention likely to work in the athlete’s situation?
Acceptability	Is the athlete and their support personnel likely to approve the intervention?
Side effects/safety	Does it have the potential to cause harm to the athlete?
Equity	Does it have the potential to isolate the athlete from others?

For practitioners who are still establishing field expertise and getting to know their athletes, there is an elevated risk of “analysis paralysis,” yet they are expected to make quick decisions based on a multitude of contextual factors that will influence their intervention. In such cases, they should seek relevant guidance from more experienced mentors and colleagues in their professional communities of practice. Furthermore, the practitioner should not be concerned about deviating from a specific behaviour change strategy or nutritional intervention if it is not accomplishing the intended goal. This flexibility in decision-making, known as “context-responsiveness,” has been demonstrated to improve outcomes in other areas of healthcare (43).

### Step 5: Audit

To keep pace with the evolving landscape of SEN, the practitioner must stay current with the latest relevant scientific literature while continuously adapting to changes in the sporting environment. Of equal importance is the practitioner’s agile commitment to their respective athlete’s ever-evolving goals, values, preferences, and behaviours. To maintain adherence to EBP principles and develop skills and knowledge in the fast-paced, ever-changing profession of SEN, practitioners must regularly reflect on the methods, applications and outcomes experienced in the field. Finding the time and self-discipline to self-audit can be challenging for many practitioners. However, those who fail to do so risk slowing their pace of professional development; continuing with ineffective approaches for engaging with Steps 1–5 of EBP; and leaning on cognitive biases, often to the detriment of their athlete when it comes to providing timely, relevant, and accurate advice. A series of questions for practitioners to self-audit their execution of the EBP steps, highlighting areas for improvement and continuous development when using the EBP framework, is provided in Supplementary material.

According to Kahneman (44), a Nobel prize recipient, the source of cognitive biases is “System 1” thinking, which refers to fast, implicit decision-making based on unconscious awareness. Because System 1 thinking results from automated cognitive processes that occur outside of awareness, a practitioner is likely to make decisions or inferences that, despite their best intentions, may contradict EBP. “System 2” thinking is the slow, deliberate, conscious processing to think and make decisions requiring effortful concentration. Reflection is a metacognitive process that engages in System 2 thinking to identify, understand, and learn from decisions made in practice. Through self-reflection, practitioners can become aware of their cognitive biases, allowing them to take note when biases surface and then slow down and participate in System 2 thinking.

It is generally accepted that there are two theoretical models of reflection, which are iterative and vertical reflection. Iterative reflection refers to a particular experience that elicits reflection, resulting in new understandings and the intention to act differently. Vertical reflection refers to reflecting in various depths, such as descriptive, intermediate, and critical (45). Kim proposed the critical reflective inquiry model developed for nursing to create and modify knowledge to respond to clinical situations and identify ineffective practices (46). Using the vertical dimension of reflective thinking, this model aims to assist practitioners in understanding the nature and meaning of their practice, correcting, and improving practice through critical self-reflection, and developing models of effective practice and theories of application. Table 4 provides an adapted version of the reflective model for practitioners to use throughout their auditing process.

**TABLE 4:** An adapted version of the critical reflective inquiry model by Kim (46).

Depth of reflection	Processes	Example questions	Products
Descriptive 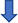	Description of events in practice, actions, thoughts, and feelings. Examination of descriptions for correctness and depth.	Describe a practice scenario that you found difficult or thought-provoking. Describe your feelings, thoughts, and actions at the time.	Descriptive narrative
Reflective 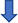	Reflective analysis of the situation Reflective analysis of intentions	How did the situation influence your actions? What values or beliefs influenced your decisions? Did you have the necessary knowledge for this situation? What were the outcomes of the situation, and were they what you intended?	Knowledge about practice processes and applications Self-awareness
Critical	Critical analysis of practice, considering the following criteria: (a) values and beliefs, (b) intentions and actions, (c) client needs, and (d) opportunities for personal growth	Were your actions in the described scenario appropriate? Were there any other outcomes you should have considered? What knowledge gaps have you identified? How do you intend to close these gaps in the future? What new insights have you gained as a result of this scenario? How might this change your practice in the future?	Learning and developing Self-critique and growth

To give accurate reflections of practice, practitioners must keep a detailed record of the nutritional interventions applied and their corresponding outcomes. Furthermore, as data collection technology advances, it is becoming easier to obtain accurate data in the field (7); hence, reflection can also act as an opportunity to evaluate any associations derived from practice. With the demand for more ecologically valid evidence in SEN, the auditing phase should serve as an opportunity for the practitioners to collaborate with researchers to identify any valid conclusions from practice, stimulating future research and moving the field of SEN onward and upward (47).

## Discussion

There are concerns that the rise of EBP could render some practitioners unduly reliant on the necessity for a scientific reference before considering a nutritional intervention, thus diminishing their inherent ability to draw on their professional experience. There is a natural tendency to regard scientific evidence as the criterion starting point that shapes decision-making within EBP. However, the triangulation of the science with the other two cornerstones (professional experience and client values) is the only way to confidently subscribe to EBP in its purest form. The scientific process is undoubtedly paramount for producing evidence with the least amount of bias that serves to influence practice; however, practitioners must remember that such evidence is merely an abstraction until it is applied to the complex, often highly uncontrolled environment of an athlete’s reality (20). This is where professional experience gained from working within a specific sport and with different athletes and understanding each athlete’s unique situation, values, and preferences come into play in successfully delivering a nutritional intervention.

Contrary to this concern, delivering a nutritional intervention that triangulates professional experience and client values with scientific evidence naturally drifts further away from the definitive literature as the recommendations become increasingly personalised to the athlete. Furthermore, if no clear evidence exists to support a nutritional intervention, the practitioner should act based on their professional judgement rooted in their understanding of the fundamental principles of SEN, current literature, experiential evidence, and ongoing, in-practice experimentation. Professional judgement can be strengthened by maintaining a detailed record of the athlete, the nutritional intervention, and associated outcomes. With advancements in data collection technology, such as wearables and real-time monitoring, collecting meaningful data from the field is becoming more accessible (7). In an ideal world, with the demand for more ecologically valid evidence in SEN, the practitioner would be working alongside researchers or as a research-active practitioner, gathering athlete/intervention records and identifying inductions from practice, which can become the focus of further experimental study, and innovation in SEN (47).

At the heart of EBP is establishing the best evidence tailored to each athlete’s unique requirements. Because of the time and effort invested in going through the five steps of EBP outlined above and the possible success when implementing the intervention with an athlete, it may be tempting to generalise a chosen intervention as the best strategy. However, the practitioner must understand that there is no such thing as a *best* strategy; instead, there are only *appropriate-at-the-time* strategies once the three cornerstones of EBP have been given equal consideration. Notably, what constitutes as best is highly subjective and varies in tandem with the athlete’s needs, training objectives, evidence, and continuous reflections. Thus, practitioners should avoid idealising their approaches and instead, in the words of the late Professor Kevin Tipton, remain “sceptical but open-minded” (60) about their approaches, constantly checking and challenging them. Although we discourage the use of idealising EBP strategies, the practitioner should have confidence in the efficacy of their approach because of going through the EBP steps. This assurance is vital in the applied environment, as some athletes or athlete-support personnel may be sceptical and require additional persuasion. Similarly, when working within a sporting organisation, confidence in the efficacy of a strategy is needed to justify the intervention to stakeholders or those who will be expected to invest in their implementation.

In SEN, there is a growing rise in self-styled “evidence-based” practitioners and the promotion of their interpretation of EBP. Given the prevalence of nutrition misinformation in the public domain, it is understandable how more practitioners want to distinguish themselves from this ilk with a title that systematically tries to remove such dogmatism. Practitioners now have access to a wealth of internet resources for obtaining scientific evidence that have been appraised, such as research reviews. Access to this information is extremely valuable for the time-pressed practitioner; however, practitioners must avoid “System 1” thinking by “appealing to authority” and blindly adhering to the practical recommendations without understanding how to apply this evidence in their athlete’s unique context (Step 4 of the EBP framework).

**FIGURE 2:**
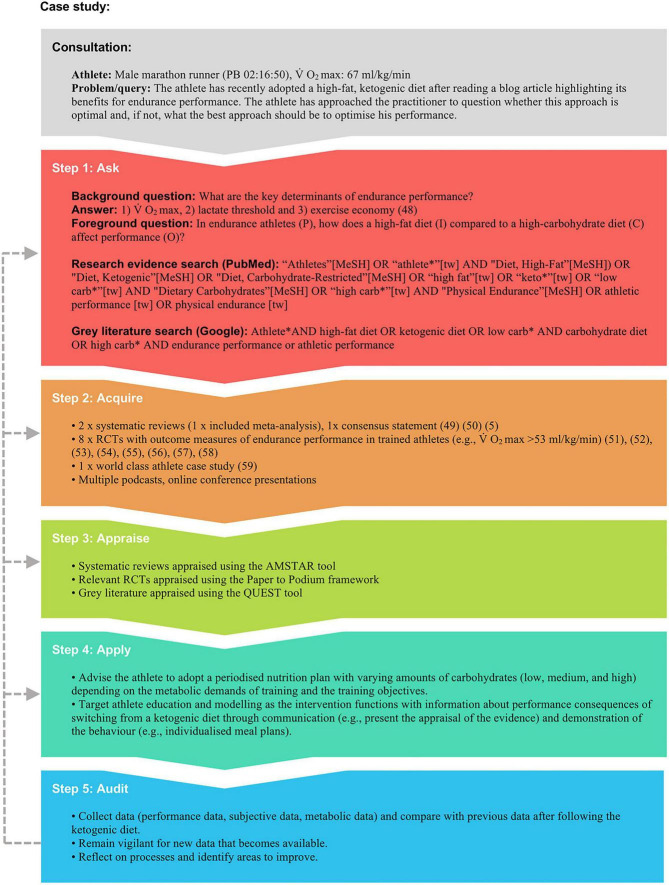
A theoretical example of how the five-step EBP framework can be applied in practice. kg, kilogram; min, minute; ml, millilitre; PB, personal best; RCT, randomized controlled trial (5, 48–59).

In the fast-paced, often time-pressed world of professional sport, our SEN-adapted framework of EBP is designed to serve as a guide, encouraging practitioners to collect the best available evidence and combine it with their professional judgement (refined through continuous reflections) and understanding of the athlete (Figure 2 provides a case study example of our framework in practice). Ultimately, the prime objective of this framework is to give athletes the best possible chance of success. Furthermore, when used correctly, the framework should provide the practitioner with a sense of assurance when delivering an intervention. This is crucial when addressing a highly contentious subject such as nutrition, particularly since the intervention may require the practitioner to convince the athlete and other stakeholders of a course of action. We recognise that the five steps are not exhaustive, and information within each step was purposefully left out to keep this a time-efficient series of prompts for the busy practitioner. In keeping with Close et al. and Coutts (8, 47), as field-based data-gathering technology improves, practitioners must become more involved in research, conducting small sample studies with similar rigour they expect when appraising research themselves. In addition to practitioners reflecting on professional competence, they should also reflect on the data gathered throughout their practice, working alongside researchers or as a research-active practitioner to identify valid research questions. The collaboration between EBP-focused practitioners and researchers working as or alongside practitioners to discover practically relevant problems for future experimentation will be what helps to bridge the often-precarious gap that exists between SEN science and practice.

## Author contributions

AR, MH, and LB drafted, critically reviewed, revised the manuscript for important intellectual content, contributed to the manuscript revision, and read and approved the submitted version. All authors contributed to the article and approved the submitted version.
