# Supplementary material for: Bridging the gap: Evidence-based practice guidelines for sports nutritionists
Source: Front Nutr. 2023 Mar 29;10:1118547. doi: 10.3389/fnut.2023.1118547 (PMC10090397; doi:10.3389/fnut.2023.1118547)
Supplement: Supplementary file 1 [file Data_Sheet_1.docx]

**Table 1.** Appraisal questions when evaluating the quality of evidence from guideline recommendations

| 1 | Are the guidelines recent and based on current evidence? |
| --- | --- |
| 2 | Do the guidelines address a clear issue? |
| 3 | Is the target demographic (e.g., trained populations) and performance outcomes clearly defined? |
| 4 | Was there a comprehensive search for and rigorous evaluation of the evidence? |
| 5 | Is there a clear link between the recommendations provided and the evidence cited? |
| 6 | Are the recommendations rated according to the strength of the supporting evidence? |
| 7 | Are all potential conflicts of interest disclosed and addressed? |

**Table 2.** An example overview of using the COM-B model and the theoretical domains framework to identify the intervention function, behaviour change techniques and mode of delivery to facilitate the target behaviour.

| **Target behaviour:** Increase intake of carbohydrate-rich foods | | | | |
| --- | --- | --- | --- | --- |
| COM-B components | **Theoretical domains framework**  Definition | What needs to happen for the target behaviour to occur?  *The athlete needs to…* | Is this a *Barrier* or *Facilitator* to the target behaviour being implemented? | Intervention functions |
| Physical capability | **Physical Skills**  An ability or proficiency acquired through practice | *have competent cooking skills to prepare meals that are rich in carbohydrates.* | B  F | Training |
| Psychological capability | **Knowledge**  An awareness of the existence of something | *understand the role of carbohydrates in endurance performance.* | B  F | Education |
| Psychological capability | **Memory, attention, and decision processes**  The ability to retain information, focus selectively on aspects of the environment and choose between two or more alternatives | *adhere to the new diet regime.* | B  F | Training |
| Psychological capability | **Behavioural regulation**  Anything aimed at managing or changing objectively observed or measured actions | *adopt a process that keeps them accountable and consistent to the new diet regime.* | B  F | Training,  Environmental  restructuring,  Enablement |
| Physical opportunity | **Environmental context and resources**  Any circumstance of a person’s situation or environment that discourages or encourages the development of skills and abilities, independence, social competence, and adaptive behaviour | *have a home and club environment that facilitates the behaviour of consuming carbohydrate-rich foods.* | B  F | Education,  Training,  Modelling,  Enablement |
| Social opportunity | **Social influences**  Those interpersonal processes that can cause individuals to change their thoughts, feelings, or behaviours | *have family, peers, and athlete support personnel that encourage the new diet regime.* | B  F | Education,  Persuasion,  Modelling |
| Reflective motivation | **Social/professional role and identity**  A coherent set of behaviours and displayed personal qualities of an individual in a social or work setting | *feel comfortable adopting a personalised diet regime.* | B  F | Education,  Persuasion,  Modelling,  Enablement |
| Reflective motivation | **Beliefs about capabilities**  Acceptance of the truth, reality, or validity about an ability, talent, or facility that a person can put to constructive use | *feel assured in their ability to adopt the new diet regime.* | B  F | Education,  Persuasion,  Modelling,  Enablement |
| Reflective motivation | **Optimism**  The confidence that things will happen for the best or that desired goals will be attained | *feel optimistic that the new diet regime will positively impact their performance*. | B  F | Education,  Persuasion,  Modelling |
| Reflective motivation | **Beliefs about consequences**  Acceptance of the truth, reality, or validity about outcomes of a behaviour in a given situation) | *feel confident that the new diet regime will lead to a positive outcome*. | B  F | Education,  Persuasion,  Incentivisation,  Coercion,  Modelling |
| Reflective motivation | **Intentions**  A conscious decision to perform a behaviour or a resolve to act in a certain way | *be fully committed to adopting the new diet regime*. | B  F | Education,  Persuasion,  Incentivisation,  Coercion,  Modelling,  Enablement |
| Reflective motivation | **Goals**  Mental representations of outcomes or end states that an individual wants to achieve | *be motivated to adopt a diet approach that has the potential to support their performance goals.* | B  F | Training,  Incentivisation,  Coercion,  Environmental restructuring |
| Automatic motivation | **Reinforcement**  Increasing the probability of a response by arranging a dependent relationship, or contingency, between the response and a given stimulus | *have internal or external incentives or reinforcements that aid their adherence to the diet regime.* | B  F | Persuasion,  Incentivisation,  Coercion,  Modelling,  Enablement |
| Automatic motivation | **Emotion**  A complex reaction pattern, involving experiential, behavioural, and physiological elements, by which the individual attempts to deal with a personally significant matter or event | *have a minimal emotional response to adopting a diet rich in carbohydrates.* | B  F | Training,  Restriction,  Environmental restructuring,  Enablement |
| **Selected intervention function(s):** | **Frequently used BCT selected** | | **Policy categories and mode of delivery** | |
| Education,  Modelling | Information about performance consequences,  demonstration of the behaviour | | Guidelines/digital media (mobile phone app) | |

**Table 3.** A performance audit of the EBP steps

| **Performance audit of EBP steps 1-5** | | **YES** | **NO** |
| --- | --- | --- | --- |
| **Step 1 - ASK** | | | |
| 1 | Am I asking foreword questions to inform my practice? | 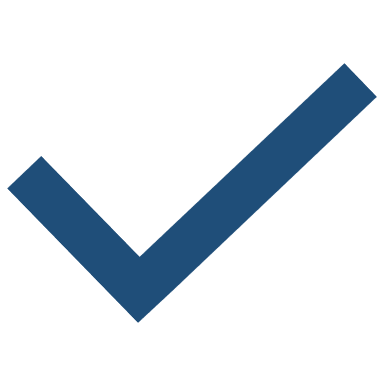 | 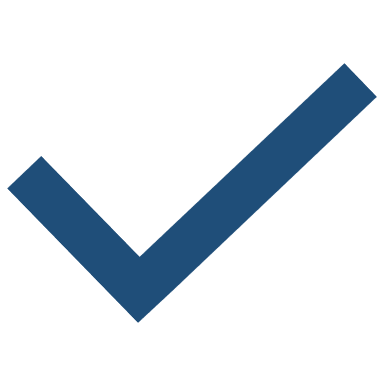 |
| 2 | Am I using the PICO structure to ask foreword questions? |  |  |
| 3 | Am I logging my questions for future reference? |  |  |
| **Step 2 - ACQUIRE** | | | |
| 1 | Am I regularly searching for the best available evidence to inform my practice? |  |  |
| 2 | Am I becoming proficient in my search for evidence (e.g., using Booleans, MeSH headings, truncations, and synonyms)? |  |  |
| 3 | Have I compared my search approach to that of other EBP practitioners? |  |  |
| **Step 3 - APPRAISE** | | | |
| 1 | Am I regularly appraising evidence? |  |  |
| 2 | Am I using the appraising tools at my disposal? |  |  |
| 3 | Am I becoming proficient in my ability to appraise evidence? |  |  |
| **Step 4 - APPLY** | | | |
| 1 | Am I proactively developing my knowledge-translation skills? |  |  |
| 2 | Am I building trust with my athletes and key stakeholders? |  |  |
| 3 | Am I tailoring my recommendations specific to the needs of each athlete? |  |  |
| **Step 5 - AUDIT** | | | |
| 1 | Am I regularly reflecting on my experiences and processes in practice? |  |  |
| 2 | Am I analysing the data collected from practice? |  |  |
| 3 | Am I acquiring the necessary skills to engage more effectively in the EBP steps? |  |  |
